# Case report: Heterogeneous mutations of SOX10 gene in a Chinese infant with Waardenburg syndrome type 4C

**DOI:** 10.3389/fped.2022.898693

**Published:** 2022-08-22

**Authors:** Suli Zhang, Shuangzhu Lin, Zhenxian Liu, Wanqi Wang, Jiayi Li, Qiandui Chen, Li Yang, Cui Wang, Qiming Pang

**Affiliations:** ^1^Department of Neuroscience, Hainan Women and Children's Medical Center, Haikou, China; ^2^First Affiliated Hospital to Changchun University of Chinese Medicine, Changchun, China; ^3^Changchun University of Chinese Medicine, Changchun, China

**Keywords:** Waardenburg syndrome type 4C, SOX10 gene, child, Hirschsprung's disease, deafness

## Abstract

A 5-month-old patient presented with grayish-blue iris bilaterally, skin and mucosal pigmentation loss, Hirschsprung's disease, full-blown growth retardation, and sensorineural deafness. The patient's whole exon gene sequencing revealed a spontaneous heterozygous code-shifting mutation in the SOX10 gene: c.803del:p.K268Sfs^*^18. The parents of the child were wild-type, and the site of the mutation is novel.

## Introduction

Waardenburg syndrome (WS) (1–3) is reported to be an auditory pigmentary disorder manifesting as a variable combination of sensorineural hearing loss (4, 5) and abnormal pigmentation of hair and skin. This syndrome is caused by abnormal proliferation, survival, migration or differentiation of neural crest-derived melanocytes, and is inherited in an autosomal dominant or recessive manner. It is a genetically heterogeneous disease. According to the different clinical symptoms, the disease is divided into four clinical subtypes, namely WS1-WS4.In this article, we report a 5-month-old patient with Waardenburg syndrome type 4C (6–8) who presented with bilateral gray-blue iris, depigmentation of skin and mucosa, Hirschsprung's disease, and sensorineural hearing loss, Whole exome gene sequencing found a transition mutation in the spontaneously heterozygous codon SOX10 (3) gene: c.803del:p.K268Sfs^*^18. And the mutation site is new.

## Case description

### Medical history

A 5-month-old boy presented with bloating and fever more than 1 month after he underwent megacolon surgery. The boy was admitted to our hospital on account of intermittent fever for 10 days. After excluding surgical contraindications, he underwent Da Vinci robot-assisted bowel adhesion surgery, appendectomy surgery, megacolon radical surgery, and internal sphincter lateral resection surgery under general anesthesia. He recovered after the operation and showed no signs of discomfort, such as bloating and vomiting, after normal feeding. The child developed a fever without any obvious precipitating causes 39 days after surgeries (2021.08.26).Other signs and symptoms included bloating and body temperature fluctuations at 36.5–37.9°C. No convulsions, cough, vomiting, diarrhea, and other symptoms were present. The child‘s condition did not improve after relatives expanded the anus and cleaned and washed the intestines. The child was admitted to hospital with acute infectious enteritis at the emergency department.

### Past medical and surgical history

Due to recurrent fever for more than 2 days, the child was hospitalized in the neonatology department of our hospital and was diagnosed as neonatal sepsis, neonatal purulent meningitis, neonatal pneumonia, neonatal lupus syndrome, common type of Hirschsprung's colon, neonatal anemia, intestinal obstruction, neonatal cholestasis, neonatal intracranial hemorrhage disease (non-traumatic), patent ductus arteriosus, atrial septal defect, and premature infant. During hospitalization, the child underwent several treatment modalities, such as invasive ventilator-assisted ventilation, blood transfusion, and anti-infection.

Two months later, the child was readmitted to the neonatal surgery department with a presentation of intermittent fever for 10 days. The child underwent Da Vinci robot-assisted intestinal adhesion surgery under anesthesia, appendectomy, megacolon radical resection, and lateral resection of the internal sphincter.

### Birth history

At a gestational age of 36 + 3 weeks (G1P1), the child was born at Dongfang People's Hospital at 05:40 on April 28, 2021. The birth weight was 2.55 kg. The amniotic fluid was clear had a volume of 500 ml at birth. No premature rupture of membranes, umbilical cord around his neck, and abnormalities in the placenta were recorded. The Apgar scores at three time points−1, 5, and 10 min—were all 10 points.

### Family history

The father had thalassemia, but the mother was healthy. No other family history of disease was recorded. Denial of consanguineous marriage.

### Physical examination

The body weight was 5 kg. The head circumference was 38 cm, and the anterior fontanelle diameter was about 0.5 ^*^ 0.5 cm. The head was flat and soft. The crying was low. The hair was yellow and sparse. The skin was white. Pigmentation loss could be observed in the systemic skin and mucosal membranes. The skin was partially fused into patches, dry, and partly accompanied by eczema. The child's head was tilted back. The limb muscle tone was high. The pupils were large and equally rounded. The reflection of light was present, and the bilateral irises were grayish blue. The pharynx was slightly hyperemic; the breath sounds in both lungs were rough. No abnormalities were identified upon cardiopulmonary examination. The abdomen was flat and soft, and the bowel sounds were about four times per minute. Capillary filling time was 2 s. The Babinski's sign was positive bilaterally, and the residual neurological physical examinations were all negative. The head was unsteady, and the child could not turn over.

### Laboratory inspection

Cardiac ultrasonography revealed that cardiac morphology and valve activity were normal. Color Doppler blood flow imaging was normal. The left ventricular systolic function indicator showed a normal value. Abdominal color ultrasonography revealed no obvious abnormalities.

The copper-protein value was 19.5 mg/dL, which was normal. Cranial MRI plain sweep + FLAIR revealed that myelination of white matter was slightly below the standard expected of children of the same age. Some extra-cervical spaces were slightly pronounced. Bilateral ventricles were slightly fuller.

The immunoglobulin, thyroid, liver, and kidney functions were normal. Additionally, electrolyte and mvocardial enzvmes were normal. The chromosomal analysis revealed a karyotype of 46, XY Hematuria metabolism screening revealed no obvious abnormalities. According to the video electroencephalography (EEG) (4 h), a mixed rhythm of 3–5 Hz was observed in the bilateral occipital areas when the patient was awake and eyes were closed. No epileptic discharges were observed during the awake phase.

Test on audio acuity showed that binaural ABR and DPOAE were not ejected. Binaural ASSR >100.

### Diagnostic assessment

More than 1 month after the megacolon surgery, the patient presented with abdominal distension with a concomitant fever of a 2-day duration.The patient had a grayish-blue bilateral iris, hypergenic expigmentation of the skin and mucous membranes, full growth retardation, and sensorineural deafness after birth. The clinical presentation of the patient first indicated a possibility of genetic metabolic disease, However, no obvious abnormalities were observed in the child's hematuric metabolic examination. A mild increase in few indicators was associated with a recent infection and nutritional support drugs. Additionally, the patient‘s history of Hirschsprung's hirschlis, gray-blue iris, and developmental delay indicated a possibility of chromosomal abnormalities, but the karyotype analysis indicated otherwise. Thereafter, the possibility of a monogenic disease was conceived.

### Further inspection

**Figure 1:**
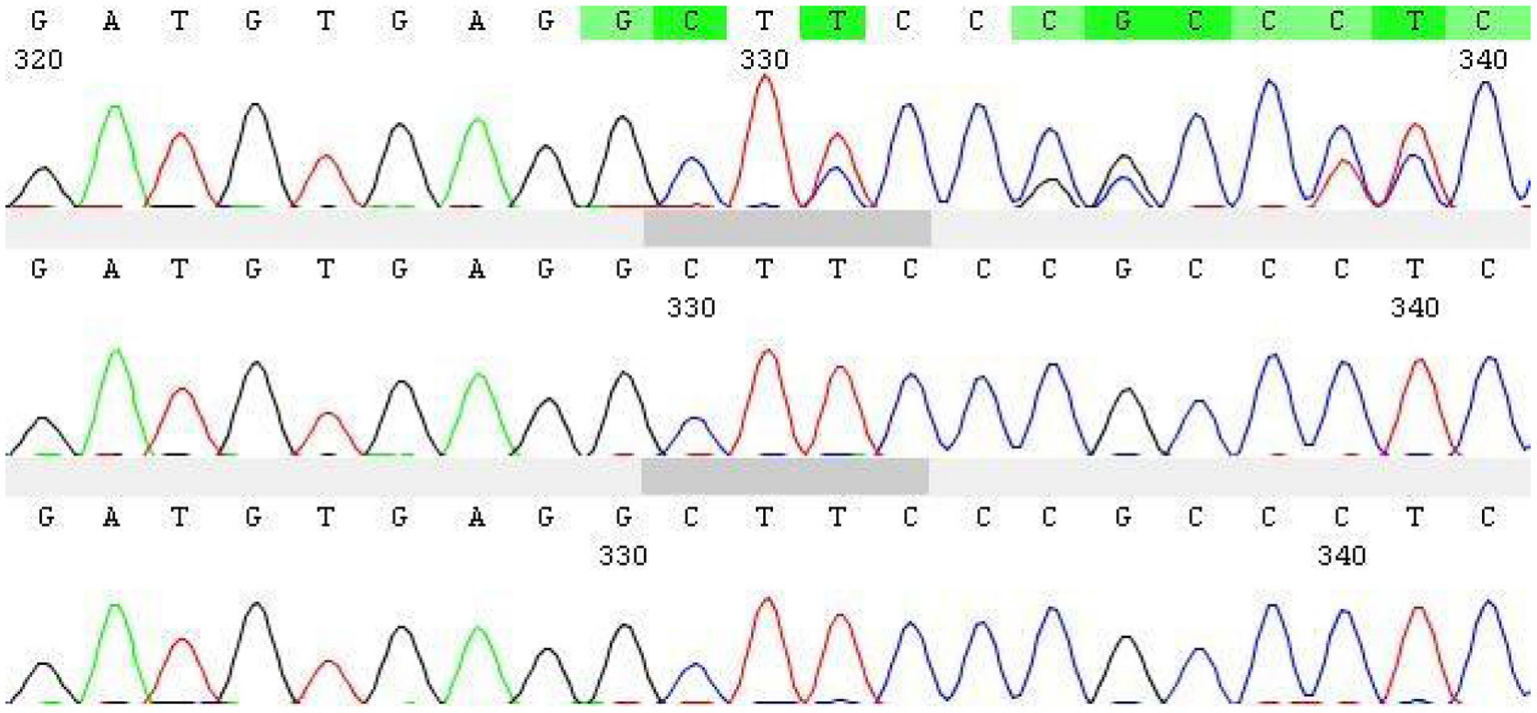
Sanger diagram.

**Figure 2:**
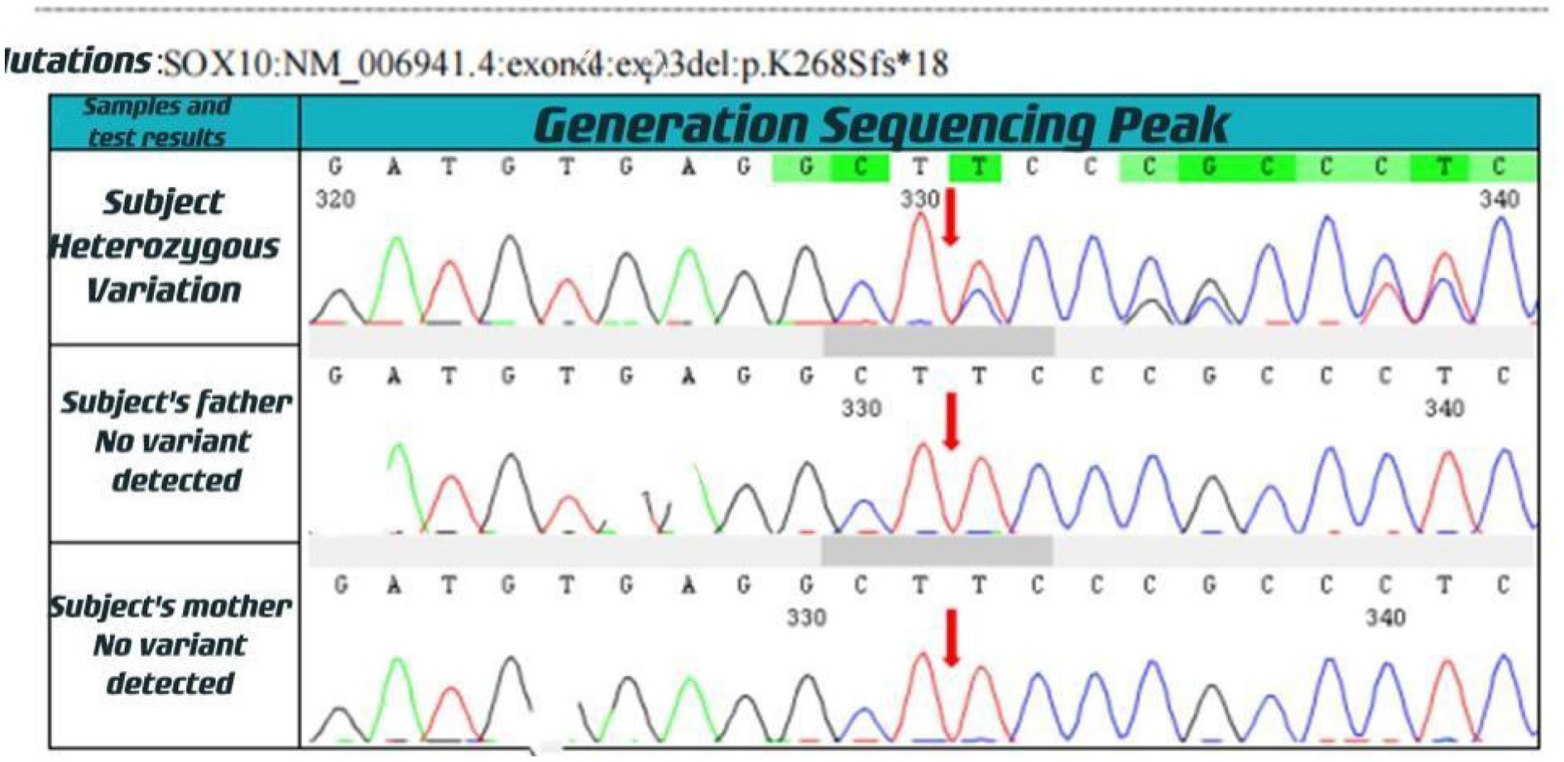
Generation sequencing peak.

**Figure 3:**
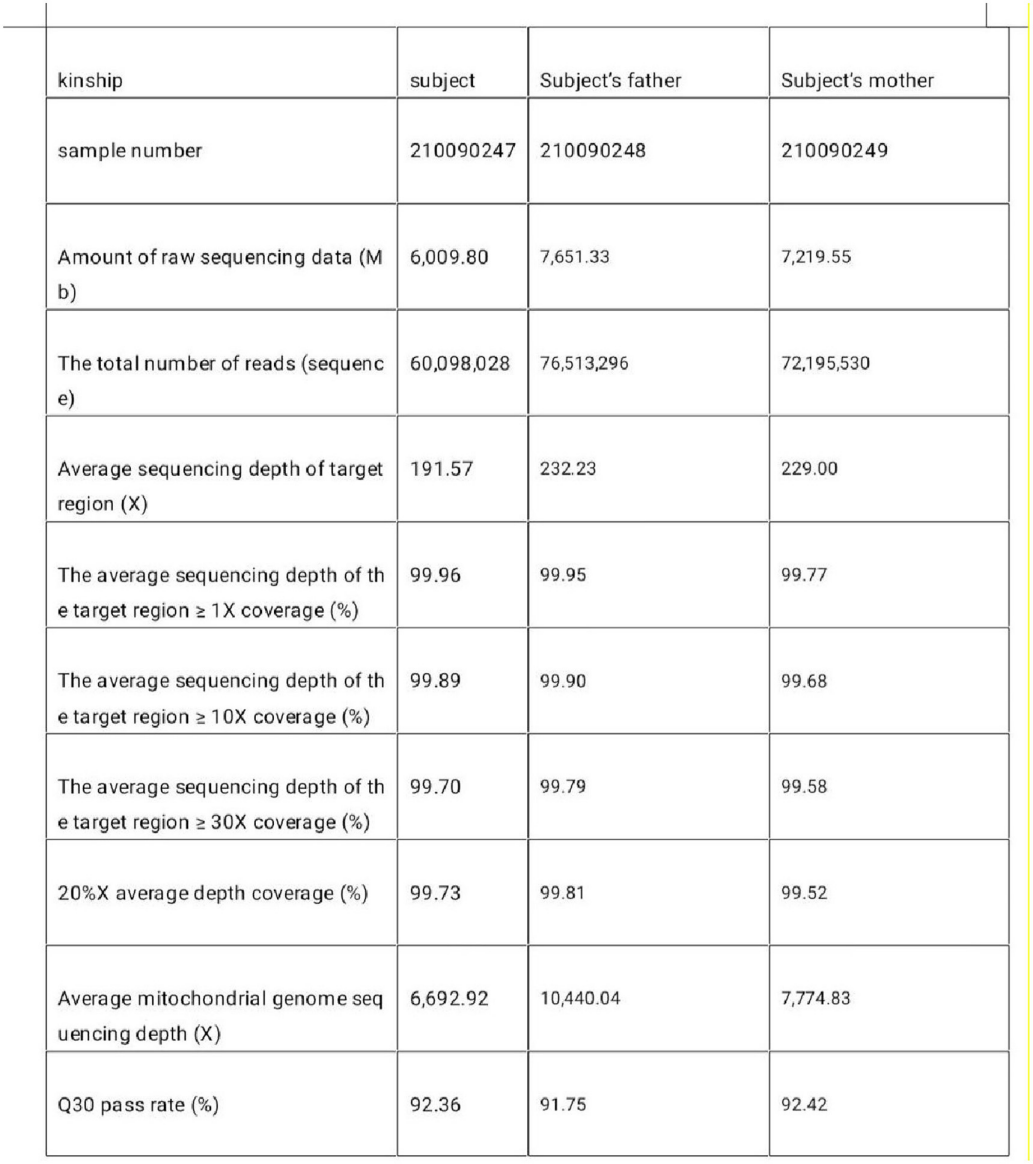
The target region of the tested sample captures the sequencing parameters.

**Figure 4:**
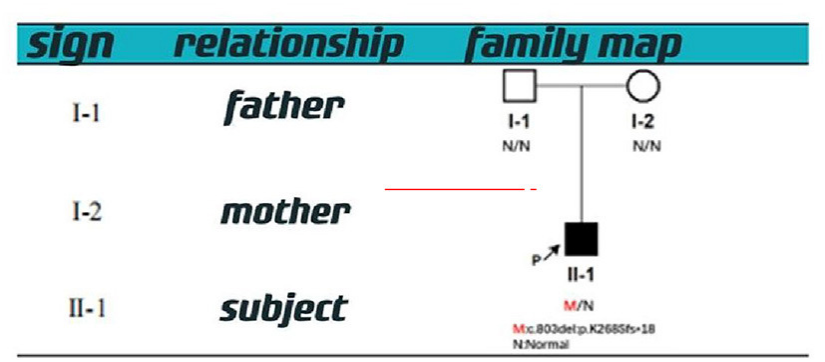
Family map.

With informed consent provided by proxy, peripheral blood samples were collected from children and parents for whole exome gene sequencing (Shanghai Vilhans). It revealed the presence of a code shift mutation, c.803del, in the SOX10 gene (NM_00694 1.4), which resulted in a variant, p.K268Sfs ^*^ 18. This variant is a low-frequency variant, according to the normal population database. No reports of this site was present in the HGMD database. The parents of the patient did not have this variant in the SOX10 gene. The mutation is nascent (Figures 1–4). This variant is pathogenic according to ACMG guidelines. The ACMG evidence PM2-Supporting described the variant as a traitor variant, which was not included in the general Dong Yaren database of gnomAD, and the classification result was pathogenic.

### Diagnostic basis

The patient‘s SOX10 gene underwent a spontaneous code shift mutation, which produced these signs: bilateral gray-blue iris, skin and mucosal visible depigmentation, congenital macrocolon, comprehensive growth retardation, and sensorineural deafness. The patient was clinically diagnosed with Waardenburg syndrome type 4C (OMIM#613266).

### Clinical history

The child was treated with anti-infective and nutritional support after admission (Piperacillin tazobactam, laoxycephalosporin, vitamin B6, and other drugs). The child had no fever, was generally in a good condition, and had stable vital signs after aggressive symptomatic treatment.

## Discussion

We performed a clinical whole exome and adjacent splicing region for gene variation analysis of the patient's family lineage and found that the patient carried a heterozygous code shift variant on the SOX10 gene: c.803del:p.K268Sfs^*^18. The second-generation sequencing and first-generation sequencing verification showed that the mutation was present and reliable. However, none of the patient's parents had the mutation, indicating that the mutation was probably new or may have resulted from germ cell chimerism in one parent. Abnormalities in the SOX10 gene can lead to autosomal dominantly inherited Peripheral demyelinating neuropathy, central myelin dysplasia, Waardenburg syndrome, Hirschsprung disease syndrome (OMIM# 609136) (9, 10), Waardenburg Syndrome Type 2E (OMIM # 611584), or Waardenburg Syndrome Type 4C (OMIM # 613266) (6–8).

This gene encodes a member of the SOX (SRY-related HMG-box) family of transcription factors involved in the regulation of embryonic development and in the determination of cell fate. The encoded protein may act as a transcriptional activator after forming a protein complex with other proteins. This protein acts as a nucleocytoplasmic shuttle protein and is important for neural crest and peripheral nervous system development.

The patient had bilateral gray-blue iris, skin and mucosal visible depigmentation, congenital macrocolon, comprehensive growth retardation, and sensorineural deafness after birth. Waardenburg syndrome (WS) is an auditory-pigmentary disorder that exhibits varying combinations of sensorineural hearing loss and abnormal pigmentation of the hair and skin.Therefore, we considered the clinical diagnosis of Waardenburg syndrome type 4C (OMIM # 613266) (1, 4, 6–11) based on the genetic results and clinical characteristics.

## Conclusion

In this study on the SOX10 gene, the code shift mutation might have caused protein truncation, which in turn led to partial loss of SOX10 function, resulting in the occurrence of WS. This study provided a comprehensive diagnosis of the child from both clinical and genetic testing aspects. New mutation sites of the SOX10 gene in genetic diagnosis were identified, which enriched the database of WS-related human gene mutations.

## Data availability statement

The original contributions presented in the study are included in the article/supplementary materials, further inquiries can be directed to the corresponding authors.

## Ethics statement

Written informed consent was obtained from the individual(s), and minor(s)' legal guardian/next of kin, for the publication of any potentially identifiable images or data included in this article.

## Author contributions

SZ: provide cases. SL: writing and analysis. ZL: financial support. WW: data sorting. JL: data sorting. QC: data sorting. QP: financial support. All authors contributed to the article and approved the submitted version.

## Conflict of interest

The authors declare that the research was conducted in the absence of any commercial or financial relationships that could be construed as a potential conflict of interest.

## Publisher's note

All claims expressed in this article are solely those of the authors and do not necessarily represent those of their affiliated organizations, or those of the publisher, the editors and the reviewers. Any product that may be evaluated in this article, or claim that may be made by its manufacturer, is not guaranteed or endorsed by the publisher.
